# Increased risk of child maltreatment and mental health problems among adolescents with restrictions regarding choice of future partner: Results from a Swedish school-based survey

**DOI:** 10.1177/14034948211053138

**Published:** 2021-10-23

**Authors:** Dennis Bengtsson, Åsa Landberg, Carolina Jernbro

**Affiliations:** 1 School of Health and Welfare, Halmstad University, Halmstad, Sweden; 2 Department of Social Sciences, Ersta Sköndal Bräcke University College, Stockholm, Sweden; 3 Division of Public Health Sciences, Department of Health Sciences, Karlstad University, Karlstad, Sweden

**Keywords:** Honour-related violence and oppression, restrictions regarding choice of future partner, child maltreatment, poly-victimisation, mental health, adolescents

## Abstract

*Background:* Honour-related violence and oppression is a violation of human rights and a public health problem. Oppression can be manifested by not being allowed to choose future partners and can increase the risk of abuse and mental health problems. *Aims:* The aim of this study was to investigate associations between restrictions regarding choice of future partner (RCP), child maltreatment and mental health problems among adolescents in Sweden. *Methods:* The study was based on cross-sectional data, including a nationally representative sample of 4741 pupils from grade nine in primary school and second year in high school. Pearson’s chi-square tests and binary logistical regression analyses adjusting for socio-demographic factors were conducted. *Results:* RCP was significantly associated to child maltreatment, including poly-victimisation, and mental health problems. In the adjusted analysis, an increased risk for all types of maltreatment, poly-victimisation (adjusted odds ratio (aOR) 10.2, confidence interval (CI) 5.6–18.4), self-harm (aOR 1.9, CI 1.1–3.2) and suicide attempt (aOR 2.4, CI 1.3–4.7) were shown in adolescents exposed to RCP compared to non-exposed. ***Conclusions:* There is an increased risk of child maltreatment including poly-victimisation and mental health problems among adolescents exposed to RCP compared to non-exposed. The study emphasizes the matter as a public health problem requiring immediate preventive measures to ensure the rights of children and adolescents.**

## Introduction

Honour-related violence and oppression (HRVO) is a global public health problem violating human rights [1]. Forced marriages are common in many parts of the world and are a form of HRVO. In 2016, an estimated 15.4 million people were in forced marriages, of which 88% of the victims were women and girls. More than one in three victims were under the age of 18 at the time of the marriage and, of these, almost half were under the age of 15 [2]. In Sweden, previous studies have shown that 5–7% of young women and 3–4% of young men have restrictions regarding choice of future partner (RCP) [3,4].

HRVO involves controlling girls’ and women’s sexuality and the heterosexual norm is central. In “honour cultures”, virginity and chastity are in focus and the family’s reputation is considered dependent on girls’ and women’s actual or alleged behaviour. This leads to restrictions in girls’ and women’s lives, including control of social life and life choices such as education, employment, marriage and divorce. The choice of partner for marriage is a family affair. Both young women and men can be victims of HRVO. Also, perpetrators of HRVO can be both men and women. The main goal is to maintain or restore the men’s and family’s honour and interests [5].

Two recent Swedish studies [6,7] conducted in four cities in Sweden highlight adolescents’ experiences regarding family’s chastity requirements and restrictions regarding RCP. Exposure to violence is one way in gaining control over the adolescent’s behaviour and future life choices. The According to one study [6], 9% of girls and 8% of boys reported concerns about RCP. Also, every fifth girl and 10th boy had chastity requirements. A small number of adolescents, with girls in majority, reported RCP in forms of forced marriage or engagement if they entered a relationship without approval from the family. Among adolescents with RCP, 62% reported exposure to one form of violence, such as physical, psychological, sexual, social, economic or material, at one point during this period. Most prominent were psychological and physical violence followed by sexual abuse and social control. Furthermore, 32% of the girls and 9% of the boys with RCP reported exposure to several types of violence (3–4 types) – that is, poly-victimisation, repeatedly over a three-year period [6]. Exposure to child abuse and particularly poly-victimisation has been associated with a range of health consequences, including attempted suicide [8,9].

Another study showed that adolescents with disabilities were particularly vulnerable, where the considered solution may be to “marry off the problem”, and that HRVO occurred regardless of socio-economic status [7].

HRVO has shown to be associated to suicidal ideations [9]. Also, feelings such as hopelessness and frustration can lead to depression [10].

Scandinavian research about HRVO among youth is limited. Previous Swedish research has focused on the prevalence of HRVO, particularly in larger cities, and adolescents’ exposure to violence in an honour setting [6,7]. However, to our knowledge, there are no nationally representative studies examining mental health consequences including self-harm, suicidal ideation or suicidal attempt for adolescents with restrictions regarding RCP. Previous research has shown the importance of RCP within the honour-related context, including exposure to different forms of violence [3,4,6]. Factors contributing to violence and ill-health among adolescents exposed to HRVO should be further investigated because of its severity and to advance in the field of research [7,11].

Thus, the purpose of the present study was to investigate the association between restrictions regarding RCP, child maltreatment and mental health problems among adolescents in Sweden. Our research questions were as follows: (1) Which sociodemographic factors are associated with RCP? (2) Is there an association between RCP and child maltreatment including poly-victimisation? (3) Is there an association between RCP and mental health problems including suicidal ideation and attempt?

## Methods

The cross-sectional data are from a Swedish national school survey on child maltreatment including 4741 adolescents, described in further detail by Jernbro and Janson [12].

### Study population and data collection

The data were collected from pupils in grade nine of primary school (14–15 years of age) and second year of high school (16–17 years of age), in autumn 2016. A sampling frame, based on the Swedish national school register consisting of 1561 primary schools and 1320 high schools, was created. The schools were stratified based on the number of pupils to ensure that schools of different sizes were included in the sampling. A random sampling of 75 schools per grade was completed, and one or two classes in each of the selected schools were chosen randomly.

Due to a high attrition rate among the invited schools, a complementary sampling of schools within the same strata was performed. The final sample consisted of 313 primary schools (grade nine) and 440 high schools (second year). Of these, a total of 142 schools participated (71 schools per grade), representing a participation rate of 22.7% of the polled primary schools and 16.1% of the high schools. There was a higher attrition rate among schools with a higher number of pupils. The most common reasons for school non-participation were lack of response on the invitation from the school management or scheduling problems. A few schools did not want to participate because of the risk of upsetting the students with the content of the survey.

A total of 273 classes took part in the study, 116 at primary school level and 157 at high school level. The pupil response rate was 82.2% in grade nine, and 75.5% in high school. The total response rate was 78.5%.

### Measures

#### Independent variable

The main variable used in the current study, applied earlier in a large Swedish study [3], was adolescents not being allowed to choose their future partner (“I get to choose who I will marry/live with as an adult”), further referred to as “*restrictions regarding RCP*”. The response alternatives *Very often true* and *Often true* were recoded to “yes”. Alternatives *Rarely true* and *Never true* were recoded to “no”.

#### Covariates

*Family economy* (“I perceive that my family can afford to buy what we need”) with the response alternatives *Very often true* and *Often true* were recoded to “*good economy*”, and *Never true* and *Rarely true* were recoded to “*poor economy*”. *Family structure* (“Which adults do you live with?”) included the response alternatives *Both my parents who live together* and *Alternately and equally with both parents*, which were recoded to “lives with both parents or alternately”; and *Mostly or only with my mother* and *Mostly or only with my mother and her new partner* were recoded to “lives mostly with one parent”. The question about *disability and chronic disease* included a list of 14 disabilities, diseases and health issues, with the answer options “no” coded to “no disability”, and “yes (diagnosed by doctor or psychologist)” and “suspected/under evaluation” coded to “diagnosed or suspected disability”.

#### Dependent variables

A detailed description of child maltreatment variables, including survey questions, items, response alternatives and recoding can be found in Table I. *Physical abuse* and *psychological abuse* (were measured with questions based on the validated instrument Conflict Tactics Scale, Parent and Child version [13], used in previous Swedish studies of child maltreatment. *Sexual abuse* was assessed using questions based on instruments from previous Swedish studies on child sexual abuse [14,15]. *Exposure to intimate partner violence*, both psychological and/or physical, was measured by questions previously used in a Swedish population-based study [16]. *Neglect* was assessed by an instrument from the Childhood Trauma Questionnaire [17] also used in the ACE-study [18]. *Child maltreatment* was considered if any of the five forms of maltreatment described above were present. *Poly-victimisation* was defined as being exposed to three to five forms of child maltreatment measured in this study.

**Table I.** Description of child maltreatment variables, including survey questions, items, response alternatives and recoding.

Variables	Survey question and items	Response alternatives	Recoding
*Physical abuse*	Has any adult done any of the following to you and, if so, how often: (1) pulled by the hair or ear, (2) smacked with an open hand, (3) hit hard with an open hand or fist, (4) kicked, (5) burned or scalded (with hot liquid), (6) grabbed by the throat/neck and choked, (7) hit with a cane, belt, ruler or similar, (8) threatened with a knife or firearm, (9) injured with a knife or firearm.	(a) Never (b) Few occasions (c) Many times	*Any physical abuse*: No: (a) on all items (1–9) Yes: (b) or (c) on any item (items 1–9) *Severe physical abuse*: (b) or (c) on items 3–9 *Frequent physical abuse*: (c) on any item
*Psychological abuse*	Has any adult done any of the following to you and, if so, how often: (1) insulted you (e.g. called you worthless, stupid, ugly), (2) locked you in the basement, a closet or in a similar confined space, (3) locked you out of the house, (4) threatened to hit or hurt you, (5) treated you as if you didn’t exist.	(a) Never (b) Few occasions (c) Many times	*Any psychological abuse*: No: (a) on all items, or (b) on item 1 and/or 5 Yes: (b) or (c) on items 2–3 or (c) on items 1 or/and 5 *Severe psychological abuse*: (b) or (c) on items 3–5
*Sexual abuse*	Has it happened that someone has done any of the following against your will: (1) showed you images of you or other people in sexual positions, on the Internet or via mobile phones, (2) asked you to perform sexual acts on the internet, (3) groped you or kissed you against your will, (4) forced you to grope or kiss someone else, (5) forced you to watch while someone else exposed his/her body or parts of his/her body to you, (6) forced you to expose your body or parts of your body, (7) forced you to have vaginal, anal or oral sex.	(a) Never (b) Few occasions (c) Many times	No: (a) on all items Yes: (b) or (c) on any item and responded that an adult was the perpetrator on the follow-up question about perpetrators.
*Neglect*	*Emotional neglect*: (1) Someone in my family helped me feel important or special, (2) I felt loved, (3) people in my family looked out for each other, (4) we felt close to each other, (5) my family was a source of strength and support. *Physical neglect*: (6) there was someone to take care of me, protect me, (7) take me to the doctor if I needed it, (8) I didn’t have enough to eat, (9) my parents were too drunk or too high to take care of me, (10) I had to wear dirty clothes.	1 = Never true 2 = Rarely true 3 = Sometimes true 4 = Often true 5 = Very often true	The responses were reversed-scored and summed, for emotional neglect (items 1–5) and physical neglect (items 6–10), respectively. For emotional neglect, respondents with scores of 15 or higher were considered neglected. For physical neglect, respondents with scores of 10 and higher were considered neglected. Any neglect was considered if the respondent had experienced emotional and/or physical neglect.
*Witnessing intimate partner violence*	Has it occurred that you have seen or heard your father (or equivalent) do any of the following to your mother (or equivalent) during your childhood: (1) used some form of physical violence (e.g. slapped her in the face, dragged her by the hair, threw things at your mother, struck with fist/a weapon, or kicked her), (2) threatened your mother with physical violence, (3) used words to insult, oppress, or dominate your mother. *The same question was repeated asking about the mother’s violence towards the father*	(a) Never (b) Few occasions (c) Many times	No: (a) on items 1 and 2, (a) or (b) on item 3 Yes: (b) or (c) on items 1 and 2, or (c) on item 3

Questions about *self-harm* (“consciously harmed myself by cutting/scratching/burning or comparable”), *suicidal ideation* (“seriously consider ending my life”) and *suicide attempt* (“attempted suicide”) have been previously used in a large Swedish population-based study [16]. The response alternative *Never* was recoded to “no”, and alternatives *1–2 times, 3–4 times* and *5 times or more* were recoded to “yes”. *Psychosomatic symptoms* were measured by the validated Psycho-somatic Problems Scale [19] including eight items (e.g. “suffered from headache”, “had difficulty concentrating”, “suffered from stomach-ache”) with the response alternatives *Never, Rarely* and *Sometimes* recoded to “no”, and *Often* and *Always* recoded to “yes” for each item. We have dichotomized the list to symptoms to “no or one to two symptoms” and “three or more symptoms”. Listwise deletion has been used to handle missing data.

### Analysis

When analysing the sociodemographic factors associated to RCP, Pearson chi-square tests were performed. To assess the associations between RCP, child maltreatment and mental health, logistic regression expressed as crude odds ratio (OR) and adjusted odds ratio (aOR) with 95% confidence intervals were performed. Covariates in the regression models included adolescents’ sex, age and disability/chronic condition, family economy and family structure. All analyses were performed in SPSS version 26 (IBM Corporation). Throughout the study, a significance level of *p* < .05 and two-tailed analysis were applied.

### Ethical considerations

The study participants were informed about the aim of the study and the terms and conditions for participation. It was made clear that participation was voluntary and that pupils had the right to stop participating any time. Parents of grade nine school pupils also received this information and were given the opportunity to deny their child to participate (if the child was under the age of 15). There was only one case in which a parent denied his/her child to participate. The pupils were informed about where they could seek help if needed, and there was an increased level of preparedness within the schools’ health services.

The study was accepted in October 2016 by the regional research ethics board in Stockholm (Dnr 2016/1014-31).

## Results

### Sociodemographic factors and restrictions regarding RCP

In the present result, 2.4% of participating adolescents reported RCP. Poor family economy, living mostly with only one parent and younger age (grade nine) were significantly associated to RCP. No significant differences were shown regarding gender (boys and girls) or disability between those who were exposed to RCP compared to non-exposed (Table II).

**Table II.** Socio-demographic factors and associations to restrictions in choice of future partner.

Socio-demographic factors	No restrictions regarding choice of future partner % (*n*)	Restrictions regarding choice of future partner % (*n*)	*p*-value
**Gender**			
Boy	50.5 (2210)	45.3 (48)	NS
Girl	49.5 (2170)	54.7 (58)	
**Family economy**			
Good economy	97.1 (4322)	85.7 (96)	< .001
Poor economy	2.9 (130)	14.3 (16)	
**Grade**			
Grade nine in primary school	47.5 (2112)	58.4 (66)	< .05
Second year in high school	52.5 (2333)	41.6 (47)	
**Family structure**			
Lives mostly with one parent	23.0 (993)	38.0 (41)	< .01
Lives with both parents or alternately	77.0 (3317)	62.0 (67)	
**Disability**			
No disability	37.4 (1560)	29.2 (28)	NS
Diagnosed or suspected disability	62.6 (2616)	70.8 (68)	

NS: non-significant.

### Restrictions regarding RCP and exposure to child maltreatment including poly-victimisation

Among the adolescents who reported RCP, 87.5% had been exposed to any child maltreatment compared to 42.2% among other adolescents. Both crude and adjusted analyses showed that RCP was significantly associated to all types of child maltreatment, as seen in Table II. Even though more girls than boys were exposed to sexual abuse (21.2% compared to 14.6%) and poly-victimisation (47.9% compared to 32.1%), the differences were not statistically significant (data not shown in table). Among the adolescents who reported RCP, 45.8% had been exposed to three or more types of violence (poly-victimisation) compared to 7.5% among other adolescents. These presented prevalences have been published earlier in a Swedish report [20]. As Table III shows, RCP was strongly associated with poly-victimisation in both the crude and adjusted analyses.

**Table III.** The association between restrictions regarding choice of future partner and child maltreatment, including poly-victimisation, expressed as odds ratios (OR) and adjusted odds ratios (aOR).^a^.

	*n*	Restrictions regarding choice of future partner OR (CI) 95%	*n*	Restrictions regarding choice of future partner aOR (CI) 95%
Neglect	4286	7.71 (4.98–11.94)***	3717	6.55 (3.78–11.35)***
Psychological abuse	4471	6.78 (4.62–9.95)***	3858	7.29 (4.45–11.94)***
Physical abuse	4342	4.52 (3.05–6.71)***	3856	4.52 (2.79–7.33)***
Witnessing intimate partner violence	4456	4.41 (2.98–6.52)***	3842	3.55 (2.10–5.99)***
Sexual abuse (adult perpetrator)	4215	3.06 (1.89–4.97)***	3638	2.73 (1.45–5.08)***
Any abuse (of the above)	4029	9.60 (5.35–17.23)***	3495	10.12 (4.93–20.79)***
Poly-victimisation	3667	10.36 (6.61–16.24)***	3220	10.19 (5.63–18.44)***

* *p* < .05.

** *p* < .01.

*** *p* < .001.

a Covariates in adjusted analysis (aOR): gender, family economy, grade, family structure and disability.

### Restrictions regarding RCP and mental health including suicidal ideation and suicide attempt

RCP was significantly associated to self-harm, suicidal ideation, suicide attempt and psychosomatic symptoms in both crude and adjusted analyses, as Table IV shows. Girls exposed to RCP reported self-harm (*p* < .001), suicidal ideation (*p* < .01) and psychosomatic symptoms (*p* < .05) more frequently than boys. There were no significant gender differences regarding suicide attempt.

**Table IV.** The association between restrictions regarding choice of future partner and mental health, including suicidal ideation and suicide attempt expressed as odds ratios (OR) and adjusted odds ratios (aOR).^a^.

	*n*	Restrictions regarding choice of future partner OR (CI) 95%	*n*	Restrictions regarding choice of future partner aOR (CI) 95%
Self-harm	4509	2.48 (1.66–3.69)***	3876	1.89 (1.11–3.22)*
Suicidal ideation	4483	2.67 (1.79–3.99)***	3856	2.37 (1.41–3.98)***
Suicide attempt	4451	4.52 (2.80–7.29)***	3827	2.44 (1.26–4.73)**
Psychosomatic symptoms (three or more)	4287	3.30 (2.25–4.92)***	3613	2.44 (1.44–4.11)***

* *p* < .05.

** *p* < .01.

*** *p* < .001.

a Covariates in adjusted analysis (aOR): gender, family economy, grade, family structure and disability.

## Discussion

This study investigated the association between RCP, child maltreatment including poly-victimisation and mental health problems in Swedish adolescents. RCP is previously shown to be an important marker within the honour-related context [3,4], related to factors concerning anxiety and child maltreatment (i.e. poly-victimisation) [6]. The present study underlines the relationship between RCP and all types of violence, specifically the poly-victimisation of adolescents exposed to RCP, as shown in a previous study [6].

Neglect, physical, psychological, material and sexual violence can have different purposes for controlling the individual’s behaviour and loyalty to the family [6,7,10], where boys and girls can have different roles in relation to exposure of RCP and violence [21].

Increased risk of self-harm, suicidal ideation, suicide attempt and psychosomatic symptoms were shown in adolescents exposed to RCP compared to non-exposed. The risk was greater for girls exposed to RCP than for boys, apart from suicide attempt where no gender difference was found. According to research, inflicting self-harm can be a strategy for processing traumatizing childhood experiences of maltreatment [7,22]. One study shows that adolescents and young adults exposed to HRVO have an increased risk to deliberate self-harm themselves severely, possibly ending up in hospitalisation [23]. Also, adverse childhood experiences and poly-victimisation are associated to symptoms such as anxiety, worry and depression [8,24].

Poor family economy, living mostly with only one parent and younger age were associated to RCP. This is in line with previous Swedish research showing that adolescents exposed to honour-related norms and restrictions tend to live in more socioeconomic segregated areas with low-educated parents and poor economy [7,25]. In this study, the association between RCP, age and family structure may be explained by the location of surveyed municipal schools and family’s socioeconomic status. The present study shows that RCP can affect the mental health of adolescents, making it an important public health matter. The present study confirms that mental health among adolescents is related to RCP, making it an important public health matter that should be further investigated. Professionals within school, healthcare and authorities need to be aware that several psychosomatic symptoms and self-harm can be signs of exposure to RCP besides exposure to child maltreatment and poly-victimisation. The school is a particularly important arena for preventive efforts since adolescents exposed to HRVO primarily turn to school personnel for support [7]. Informing children and adolescents about their rights, and where, when and how they can seek support and protection is central. Educational efforts are needed to ensure that professionals who meet children and adolescents have the knowledge required to identify HRVO and the ability to support and protect.

### Methodological considerations

#### Strengths and limitations

This is the first nationally representative study in Sweden on the association between RCP, maltreatment and mental health problems, including a large sample size and high response rate among adolescents. Choice of life partner is fundamental in the honour-related context [1,6,7,10,23,25]. However, it cannot be assured that all adolescents who reported RCP live in an honour-related context. A complementary question regarding chastity requirements, besides RCP, could have been beneficial in clarifying the damaging nature of the honour-related context further. In addition, the surveyed question concerning RCP is a subjective measure about the adolescent perception as controlled, or not, regarding RCP.

In our study, few reported RCP compared to other Swedish studies conducted in larger cities [7,25]. This difference could be due to the higher attrition rate among larger schools (i.e. schools in metropolitan areas) in the present study. The relatively small number of adolescents reporting RCP limited the possibilities for specific analyses. For example, poly-victimisation as a potential moderator in the association between RCP and mental health problems could not be investigated due to this limitation. Further research on RCP in relation to poly-victimisation and mental health problems, including a larger sample size, would be preferable in additional understanding of the subject. A larger sample size could also clarify some of the insignificant results regarding gender differences.

Because of the cross-sectional design of the present study, causal associations cannot be ascertained. The inclusion of relevant socio-demographic factors in the adjusted analysis may present a more reliable picture of the context. However, the missing data for the disability variable was almost 8%, which resulted in losing parts of the sample in the adjusted analyses. The results need to be interpreted with caution due to loss in statistical power. Otherwise, missing data for most key variables were low. Furthermore, memory bias and underreporting due to perceived feelings such as shame and guilt in relation to the subject can occur. Exposure to child maltreatment is considered traumatizing and whereas children have the ability of recollection, many do not want to report the event [26].

## Conclusions

Adolescents restricted regarding choice of partner are at an increased risk of child maltreatment including poly-victimisation, self-harm, suicidal ideation, suicide attempt and psychosomatic symptoms compared to other adolescents. This study confirms the matter as an important public health problem requiring immediate actions, such as early interventions to prevent HRVO, increased recognition of HRVO among professionals and enhancing the support for the exposed.
